# Aboveground Biomass Mapping and Analysis of Spatial Drivers in the Qinghai–Xizang Plateau Permafrost Zone: A Case Study of the Beilu River Basin

**DOI:** 10.3390/plants13050686

**Published:** 2024-02-29

**Authors:** Yamin Wu, Jingyi Zhao, Ji Chen, Yaonan Zhang, Bin Yang, Shen Ma, Jianfang Kang, Yanggang Zhao, Zhenggong Miao

**Affiliations:** 1 National Cryosphere Desert Data Center, Lanzhou 730000, China; 2 Beiluhe Observation and Research Station of Frozen Soil Engineering and Environment, State Key Laboratory of Frozen Soil Engineering, Lanzhou 730000, China; 3 Northwest Institute of Eco-Environment and Resources, Chinese Academy of Sciences, Lanzhou 730000, China; 4 Middle Yarlung Zangbo River Natural Resources Observation and Research Station of Tibet Autonomous Region, Research Center of Applied Geology of China Geological Survey, Chengdu 610036, China; 5 Key Laboratory of Natural Resource Coupling Process and Effects, Beijing 100000, China

**Keywords:** aboveground biomass, Qinghai–Xizang Plateau, random forest, geodetector, alpine grassland, permafrost, Beilu River Basin

## Abstract

Aboveground biomass (AGB) serves as a crucial measure of ecosystem productivity and carbon storage in alpine grasslands, playing a pivotal role in understanding the dynamics of the carbon cycle and the impacts of climate change on the Qinghai–Xizang Plateau. This study utilized Google Earth Engine to amalgamate Landsat 8 and Sentinel-2 satellite imagery and applied the Random Forest algorithm to estimate the spatial distribution of AGB in the alpine grasslands of the Beiliu River Basin in the Qinghai–Xizang Plateau permafrost zone during the 2022 growing season. Additionally, the geodetector technique was employed to identify the primary drivers of AGB distribution. The results indicated that the random forest model, which incorporated the normalized vegetation index (NDVI), the enhanced vegetation index (EVI), the soil-adjusted vegetation index (SAVI), and the normalized burn ratio index (NBR2), demonstrated robust performance in regards to AGB estimation, achieving an average coefficient of determination (*R*^2^) of 0.76 and a root mean square error (*RMSE*) of 70 g/m^2^. The average AGB for alpine meadows was determined to be 285 g/m^2^, while for alpine steppes, it was 204 g/m^2^, both surpassing the regional averages in the Qinghai–Xizang Plateau. The spatial pattern of AGB was primarily driven by grassland type and soil moisture, with q-values of 0.63 and 0.52, and the active layer thickness (ALT) also played a important role in AGB change, with a q-value of 0.38, demonstrating that the influences of ALT should not be neglected in regards to grassland change.

## 1. Introduction

The Qinghai–Xizang Plateau, the world’s highest and most extensive high-altitude permafrost region, is dominated by alpine grasslands, with a 54–70% land cover [1]. These ecosystems play vital roles in global carbon sequestration, water regulation, and biodiversity [2]. The aboveground biomass (AGB) serves as a critical indicator of grassland productivity, energy flow, and material cycling [3,4,5]. Understanding the spatial patterns and influencing factors of AGB is crucial for assessing the health and resilience of these ecosystems under changing environmental conditions [6,7].

Remote sensing technology offers an efficient and economical tool for AGB estimation [8,9,10]. While previous studies of the Qinghai–Xizang Plateau relied on coarse-resolution MODIS data (250–500 m) [11,12], limiting their ability to capture fine-scale variations, high-resolution options like Landsat 8 OLI (30 m) and Sentinel-2 (10 m) are becoming increasingly accessible and computationally feasible [13,14,15]. These finer resolutions can better correspond to field-measured AGB data, significantly improving estimation accuracy [16]. Vegetation indices (e.g., NDVI, EVI, SAVI, RVI, NBR) have been successfully used as predictors for AGB estimation [17,18,19]. The random forest (RF) algorithm, known for its ability to handle complex, high-dimensional data, has proven effective and robust for grassland AGB estimation [20,21,22]. Its ensemble approach of building multiple decision trees enhances both accuracy and stability [12,22], rendering the RF model particularly suitable for this investigation.

The spatial distribution of aboveground biomass (AGB) within alpine grasslands is governed by a multifaceted interplay of environmental determinants, including vegetation type, soil characteristics, and climatic parameters such as precipitation, temperature, and snow cover [23,24,25,26]. Additionally, in the context of the Qinghai–Xizang Plateau’s permafrost region, specific permafrost characteristics like freeze-thaw dynamics, the thickness of active layer, and permafrost temperature play a significant role in regulating vegetation growth [27,28,29]. Understanding these interactions and their impact on AGB is crucial for predicting future changes and informing effective management strategies for maintaining grassland productivity and ecological integrity.

This study endeavors to elucidate the spatial distribution and underlying driving forces of AGB in the Beilu River Basin, a representative region within the Qinghai–Xizang Plateau hinterland. Employing the RF model, we construct an AGB estimation model by integrating data from 140 field sampling points and high-resolution Landsat 8 and Sentinel-2 data accessed through Google Earth Engine (GEE). Furthermore, we apply the geodetector tool to identify the key factors, along with their interactions, influencing AGB distribution. The study’s objectives encompass: (1) mapping the spatial pattern of AGB across the alpine grasslands in the Beilu River Basin; (2) identifying the main factors, along with their interactions, influencing AGB distribution.

## 2. Materials and Methods

### 2.1. Study Area

**Figure 1:**
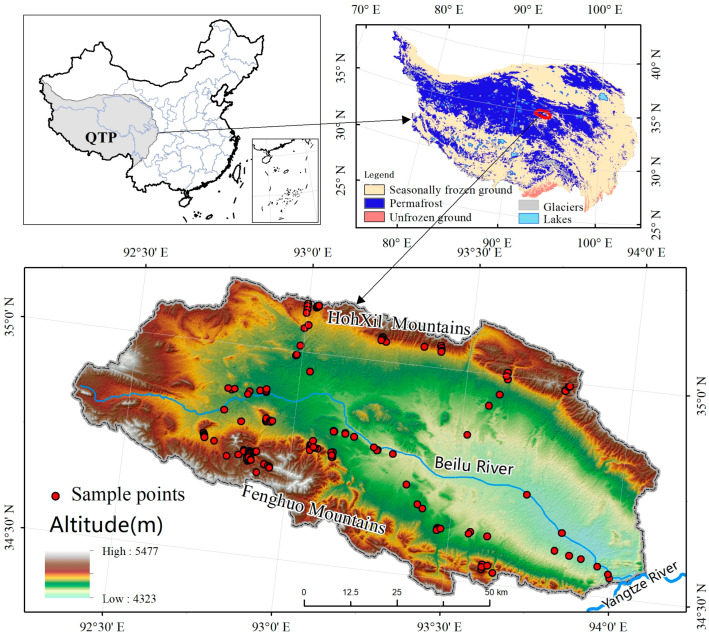
Location of the Beilu River Basin and the AGB sampling point.

The Beiliu River Basin, situated in the Yushu Tibetan Autonomous Prefecture of Qinghai Province, constitutes a sub-basin of the Yangtze River Headwaters on the Qinghai–Xizang Plateau (Figure 1). The basin spans an approximate area of 8000 km^2^ and has a plateau continental climate dominated by a westerly circulation. It is subject to prolonged, cold, and dry winters, in contrast to brief, short, mild, and moist summers. The mean annual air temperature fluctuates between −8 °C and −2 °C, with annual precipitation ranging from 300 mm to 500 mm. According to the data from Zou et al. (2017) [30], the permafrost area covers 78% of the total basin area. The basin comprises typical alpine grassland ecosystems, including alpine swamp meadows, alpine meadows, alpine steppes, alpine desert steppes, and alpine deserts. The dominant plant species across these different grassland types are detailed in Table 1, including *Kobresia species, Carex moorcroftii, Stipa purpurea, Leontopodium pusillum, Arenaria qinghaiensis, Androsace tapete, Rhodiola, and Saussurea tangutica.*

### 2.2. Data Sources

#### 2.2.1. Collection of AGB Data

Field surveys were conducted from late July to mid-August, 2022, to obtain the grassland AGB field sample data, which consisted of 140 sample points distributed across the study area. These sample plots were chosen to encompass the diversity of the grassland types, soil types, and terrain topography in the area. The sampling information included longitude, latitude, altitude, slope, aspect, vegetation coverage, grassland community species, and vegetation height. Within each sample plot, six sub-sample quadrats (20 cm × 20 cm) were randomly located at least 5 m apart from each other in each sample plot. All the plant specimens in the sub-sample quadrats were harvested, bagged, and weighed in the field, and subsequently transported to the laboratory for dry weight measurement. These samples were dried in an oven at 65 °C for 48 h and then reweighed to calculate the moisture content and the dry biomass. The AGB for each sample plot was estimated by averaging the values from the six sub-sample quadrats. Table 1 shows detailed information on the AGB data for the different grassland types.

#### 2.2.2. Climate Data

**Figure 2:**
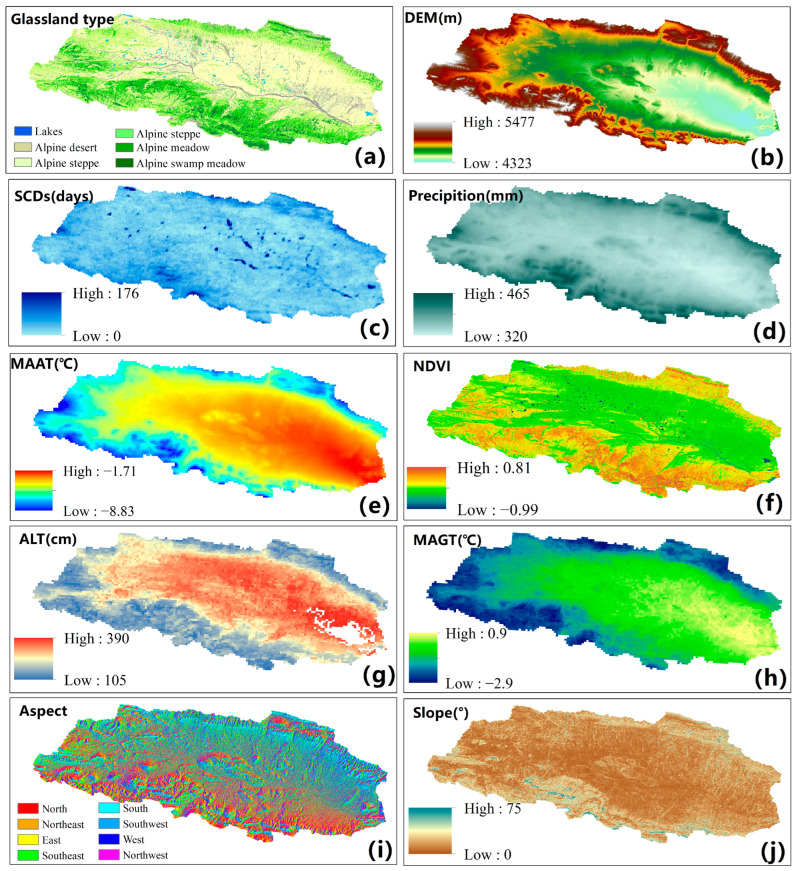
The input environmental factors for the geodetector tool (**a**) Glassland type; (**b**) DEM; (**c**) Snow cover days (SCDs); (**d**) Annual precipition (Precipition); (**e**) Mean annual air temperature (MAAT); (**f**) Normalized differnce vegetation index (NDVI); (**g**) acitve layer thcikness (ALT); (**h**) Mean annual ground temperature (MAGT); (**i**) Slope direction (Aspect); (**j**) Slope gradient (Slope).

This study evaluates the impact of climatic variables on AGB, incorporating climate data as a key variable for the geodetector model. The climate data consists of the mean annual air temperature (MAAT) and the annual precipitation (AP) at a spatial resolution of 1 km for the year 2022 [31,32], along with the 500 m resolution snow cover days for 2021 [33]. The MAAT (Figure 2e) data source is the annual mean temperature data at 1 km resolution in China (1982–2022), and the AP (Figure 2d) data source is the annual precipitation data at 1 km in China (1982–2022), which were sourced from the National Earth System Science Data Center. The snow cover days (Figure 2c) data source is the remote sensing monitoring data set of snow days in Qinghai Province from 2002 to 2021, sourced from the National Cryosphere Desert Data Center (NCDC).

#### 2.2.3. Terrain Data

The topographic factor is another important driver affecting the spatial distribution of grassland AGB. This study utilizes topographic factors to analyze their influence on AGB and as the inputs both for the RF model and geodetector. A 30 m resolution digital elevation model (DEM) (Figure 2b) from the Shuttle Radar Topography Mission (SRTM), provided by the U.S. Geological Survey (USGS), is employed to calculate the topographic parameters such as the slope gradient (Figure 2j) and slope direction (Figure 2i), which reflect the influence of topography on hydrothermal conditions and vegetation distribution.

#### 2.2.4. Vegetation Types, Soil Moisture, and Frozen Soil Data

Grassland classification maps (Figure 2a), at 30 m resolution with a kappa coefficient of 90%, were obtained on the GEE platform by employing the random forest classification method, using 140 AGB sampling points, 75 manual classification points, and Landsat images. The grasslands were mainly categorized into alpine swamp meadows, alpine meadows, alpine steppes, alpine desert steppes, and alpine deserts. The mean soil moisture data (Figure 2f) for July–August, 2022, was replaced by the mean value of the July–August 2018–2020 1 km daily soil moisture dataset over the Qinghai–Tibet Plateau (2001–2020), which was provided by the National Tibetan Plateau Data Center (TPDC) (https://data.tpdc.ac.cn/zh-hans/data/b611fb43-c18c-4966-a85c-949ce1ca60f2 (accessed on 21 November 2023)).

The frozen soil data, including the active layer thickness for permafrost (ALT, Figure 2g) and the mean annual ground temperature data (MAGT, Figure 2h), were obtained from the Current State and Past Changes in Frozen Ground at the Third Pole on the National Tibetan Plateau/Third Pole Environment Data Center [34].

### 2.3. Methods

#### 2.3.1. Random Forest Model Based on the GEE Platform

This study applied an RF algorithm based on the GEE platform, using 140 field measurements of AGB as training samples, Landsat 8 OLI and Sentinel-2 imagery data from June to September 2022 as input data, and 9 VIs (NDVI, EVI, SAVI, DVI, RVI, GNDVI, GRVI, NBR1, NBR2, Table 2) and three topographic factors (elevation, slope, and aspect) as independent variables. The aim was to develop an RF model for estimating the AGB of alpine grassland in the Beilu River Basin. The model divided the training and testing sets randomly using a 0.8:0.2 ratio. The steps for constructing and applying the RF model were as follows:

(1) Select the optimal combination of Landsat 8 OLI and Sentinel-2 images from June to September 2022 from the GEE platform, based on the criteria for cloud cover and temporal and spatial consistency;
(2) Perform pre-processing operations on the remote sensing images, including atmospheric correction, radiometric correction, and geometric correction, to extract various VIs;
(3) Spatially match the VIs with the field measurements of the AGB to obtain training and validation samples;
(4) Establish a regression model between the AGB and VIs using the RF algorithm and evaluate the model performance using the coefficient of determination (*R*^2^) (Equation (1)) and the root mean square error (*RMSE*) (Equation (2)).

(1) $$R^{2}=1-\frac{\sum_{i=1}^{n}{\left(\hat{y_{i}}-y_{i}\right)}^{2}}{\sum_{i=1}^{n}{\left(\hat{y_{i}}-\bar{y_{i}}\right)}^{2}}$$

(2) $$RMSE=\sqrt{\frac{\sum_{i=1}^{N}{\left(\hat{y_{i}}-y_{i}\right)}^{2}}{n}}$$

where *n* denotes the number of sample plots, $y_{i}$ and $\hat{y_{i}}$ represent the measured and estimated AGB values, respectively, and $\bar{y_{i}}$ is the mean of the AGB measurements.

#### 2.3.2. Geodetector

The influence of multiple factors on a spatially targeted variable, as well as the interactions between different factors, can be analyzed using geodetector, a statistical method based on spatial stratified sampling. In the Beilu River Basin, the influences of grassland types, soil moisture, climatic factors (MAAT, precipitation, and SCDs), permafrost factors (ALT, MAGT), and topographic factors (DEM, slope, and aspect) (Figure 2) on AGB during the 2022 growing season, as well as the interactions between these factors, were quantitatively analyzed using the geodetector method. The Geodetector package in R (https://cran.r-project.org/web/packages/geodetector/index.html (accessed on 25 November 2023)) was used to perform the geodetector analysis in the study area. Before performing the geodetector technique, the 10 environmental variables and the estimated AGB would first be uniformly resampled to a resolution of 1 km.

## 3. Results

### 3.1. Random Forest Model Development and Validation in GEE

**Figure 3:**
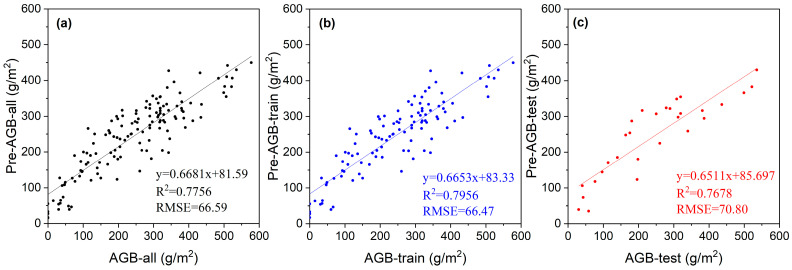
The correlation between the estimated and measured AGB values based on Model 8. (**a**) all samples, n = 140; (**b**) train set, n = 111; (**c**) test set, n = 29.

After partially combining the 9 VIs, 11 models were obtained, which were then evaluated by the *R*^2^ and *RMSE* of the test set; their *R*^2^ generally ranged from 0.60–0.8, and the *RMSE* ranged from 65–89 g/m^2^ (Table 3). These results indicate that the models effectively captured the AGB dynamics in the study area, with minimal systematic error. From the comparison of the models in Table 3, it can be seen that Model 8, based on the training set showed the best performance with the highest *R*^2^ of 0.7956 and the *RMSE* could be as low as 66.47 g/m^2^. Figure 3 illustrates the correlation between the estimated and the measured AGB values for all samples, the training set, and the test set, using Model 8.

We estimated the AGB in the Beilu River Basin using an RF regression model in the GEE and analyzed the impact of various VI combinations on the performance of the RF model from training set. The results showed that EVI and NDVI were the most important predictor variables, contributing significantly to the estimation of AGB. NBR2 and SAVI were also important characteristic variables, and the *R*^2^ of the model was still as high as 0.75 when only these four VIs were used; the simulation performance of the most of model would be slightly improved when other VIs were added. The optimal model performance was attained by incorporating six VIs: NDVI, EVI, SAVI, DVI, NBR1, and NBR2, which yielded the highest *R*^2^ of 0.7956 and the low *RMSE* of 66.47 g/m^2^. Conversely, the inclusion of GRVI and GNDVI indices had a detrimental effect on the model’s performance, resulting in a decrement of *R*^2^ by 0.04 and 0.07, respectively.

### 3.2. Spatial Distribution of Grassland AGB in the Beilu River Basin

**Figure 4:**
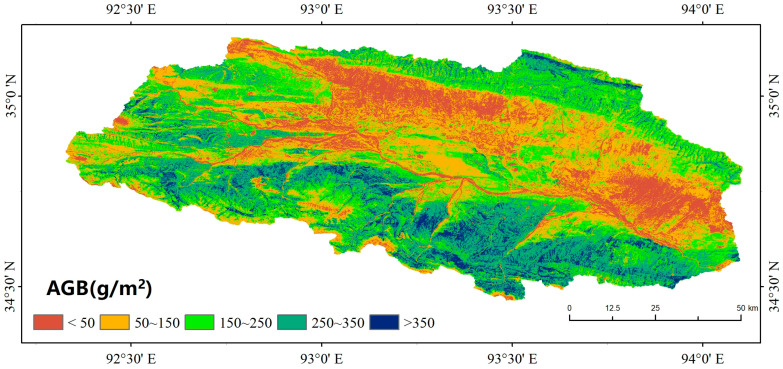
The spatial pattern of grassland AGB in the Beilu River Basin during the 2022 growing season.

Utilizing the RF model developed in Section 3.1, we generated a spatial distribution map of AGB in the Beilu River Basin in the growing season (July–August) of 2022 (Figure 4). Figure 4 revealed that the AGB of alpine grassland in the Beilu River Basin exhibited significant spatial variability, generally following a pattern of high values on the south bank and low values on the north bank of the Beilu River. The highest AGB values (>250 g/m^2^ on average and >450 g/m^2^ at maximum) occurred in the mid- and high-mountainous areas on the northern slopes of the Fenghuo Mountains and in the high mountainous regions on the southern slopes of the HohXil Mountains. The dry plains along the north bank of the Beilu River exhibited the lowest AGB, with mean and minimum values ranging from 50–100 g/m^2^ and below 50 g/m^2^, respectively. The spatial distribution of AGB in the study area is influenced by various factors, such as climatic conditions, soil moisture, and vegetation types.

### 3.3. Driving Factors of Vegetation in the Beilu River Basin Based on the Geodetector Method

In this paper, 10 factors (precipitation, MAAT, ALT, MAGT, SCDs, soil moisture, vegetation types, DEM, slope, and aspect) (Figure 2) were selected to quantitatively analyze the drivers of AGB in the Beilu River Basin. The explanatory power of the factors in regards to AGB was determined by the q-value.

**Figure 5:**
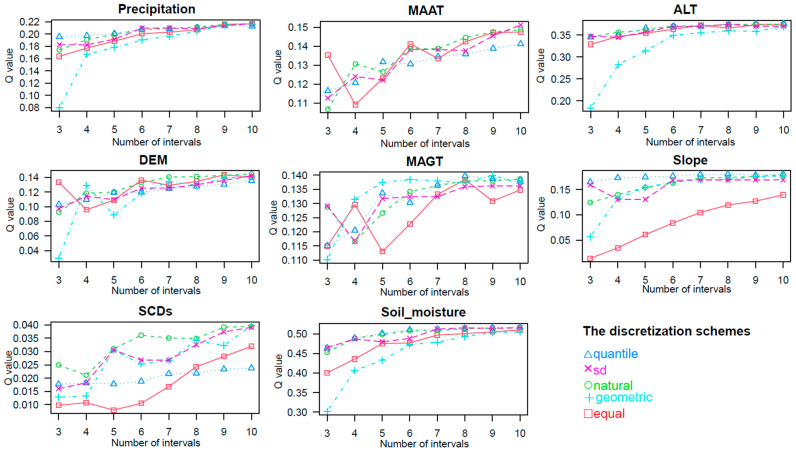
Effect of different discretization schemes on q-value.

**Figure 6:**
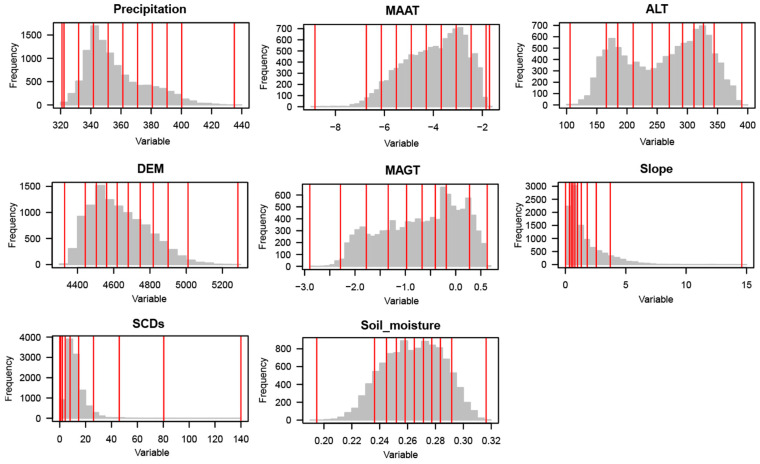
Categorical ranges of continuous variables. The gray bars represent the frequency distribution of the data, while the red dividing lines represent the interval positions of variable stratification.

The input variables of the geodetector method are type variables, and all eight factors, except for the aspect and vegetation types, are continuous variables, so the continuous variables need to be optimally discretized before using factor detection. The Geodetector package based on R language provides five discretization methods (sd, equal, natural, quantile, and geometric) to optimally discretize the eight factors, and the numbers of breakpoints are set between 2 and 10. The effect of different discretization methods and breakpoint schemes on the q-value is shown in Figure 5 and Figure 6. As the classification interval increases, the q-value shows a gradual increase, and different breakpoints schemes have varying effects on the q-value. The discretization algorithm and breakpoint numbers corresponding to the biggest q-value are chosen to ensure that the explanatory power of the factor for AGB is maximized. All variables were categorized into 10 categories, except for the MAAT, which was categorized into 9 categories; “sd”, “natural”, “quantile”, and “geometric” were selected as the most optimized discretization, as shown in Table 4.

**Figure 7:**
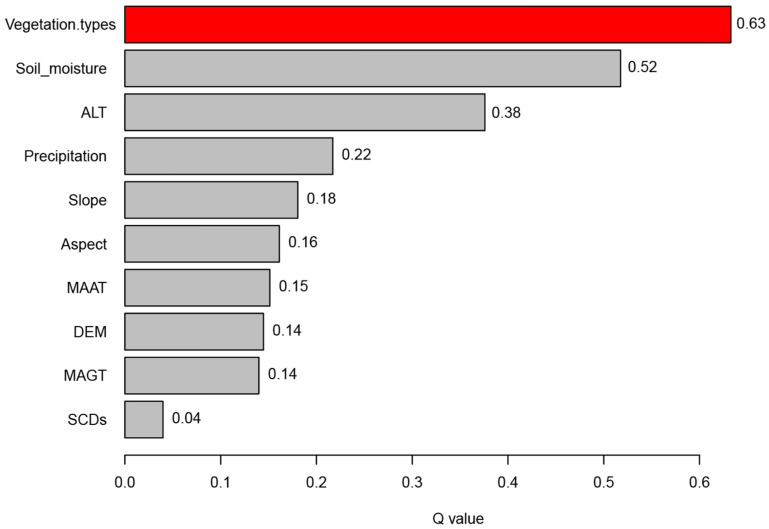
The q-value of each driving factor for AGB. The red bar represents the highest q-value.

The geodetector factor detection method was applied using the 10 factors as independent variables and AGB as the dependent variable to assess the degree to which each factor explains AGB. The results indicated that all factors had significant (*p* < 0.05) effects on AGB (Figure 7). The q-values, in descending order, were vegetation types (0.63), soil moisture (0.52), ALT (0.38), precipitation (0.22), slope (0.18), aspect (0.16), MAAT (0.15), DEM (0.14), MAGT (0.14), and SCDs (0.04). Vegetation types exhibited the strongest explanatory power for AGB, while SCDs showed the weakest effect. Overall, vegetation types and soil moisture are the primary contributors to AGB, with q-values exceeding 0.5. The q-values of ALT, precipitation, slope, aspect, MAAT, DEM, and MAGT fell within the range of 0.1 to 0.5, suggesting a weak influence on AGB.

**Figure 8:**
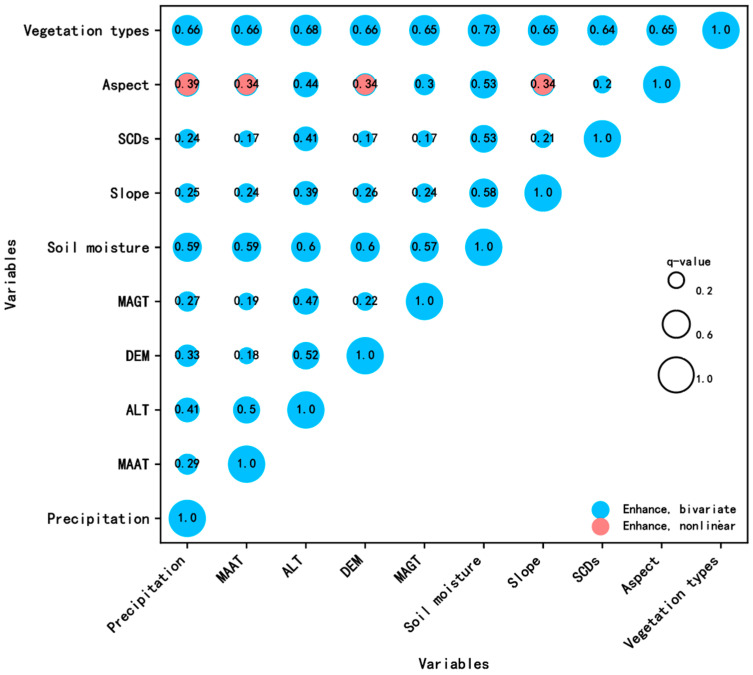
Interactive detection of different driving factors.

Geodetector factor interaction detection was used to assess the effect on AGB in the case of two factors interacting together. The results show that the explanatory power for AGB after the two-factor interaction is higher than that of the one singlefactor effect (Figure 8). The type of interaction is dominated by two-factor linear enhancement, except for aspect’s interaction with four factors, which is nonlinearly enhanced. The explanatory power of AGB increased after the 10 factors interacted with each other, with vegetation types and soil moisture interaction having the highest explanatory power (0.73), which suggests that the interaction of vegetation types and soil moisture has a greater impact on AGB.

## 4. Discussion

### 4.1. Comparison between Estimated AGB and the Optimal Vegetation Indices Model

We applied an RF model to estimate and map AGB of grasslands in the Beilu River Basin at a high spatial resolution of 30 m. The model’s performance was evaluated using *R*^2^ and *RMSE*, revealing a high accuracy, with an *R*^2^ and *RMSE* of 0.78 and 66.59 g/m^2^, respectively (Figure 3a), which were comparable to the results from the large-scale and coarse-resolution simulations. This confirmed the rationality of the high-resolution AGB mapping of our RF model in this region. Based on the RF model, we estimated the mean AGB of the grasslands in the study area for the 2022 growing season to be 180.51 g/m^2^, with alpine meadow and alpine grassland averaging 285.06 g/m^2^ and 204.84 g/m^2^, respectively. These values exceed most reported measurements and estimates for alpine grasslands in the Qinghai–Xizang Plateau [49,50]. One possible explanation for this discrepancy is that our field sampling, conducted from July 15 to August 15, coincided with the period when AGB is near its annual peak [51]. Additionally, the relatively low grazing intensity in our study region compared to other grasslands in the Qinghai–Xizang Plateau [1,52] may have contributed to the higher AGB values. Furthermore, we also observed that alpine grasslands, despite having a lower vegetation cover, exhibited higher plant heights and larger leaf areas, which could contribute to the higher AGB values [53,54]. Notably, over 50% of our samples had AGB values exceeding 250 g/m^2^ (Table 1), which increased the weight of the high AGB values in the RF model.

We evaluated the performance of the RF models with varying combinations of vegetation indices (VIs) for AGB estimation in the Beilu River Basin using a test set. The *R*^2^ and *RMSE* values indicated that the RF models relying solely on NDVI or EVI achieved *R*^2^ values above 0.6 (Table 2) and *RMSE* values around 90 g/m^2^, indicating that NDVI and EVI were the most important and influential VIs for AGB estimation [55], which again validates the correctness of numerous previous related studies using NDVI to estimate AGB [11,56]. The addition of NBR2 or SAVI to the vegetation indices combination significantly improved the prediction accuracy, as both NBR2 and SAVI can mitigate soil background effects and are more suitable for sparse vegetation cover areas [57,58]. However, an increase in the number of VIs did not consistently enhance the *R*^2^ values. The optimal combination, consisting of EVI, NDVI, NBR2, SAVI, DVI, and RVI, achieved an *R*^2^ value of 0.7896 and an *RMSE* value of 64.9 g/m^2^. However, adding GNDVI and GRVI to the model reduced the *R*^2^ value slightly, possibly because GNDVI and GRVI exhibited lower values than NDVI and showed less ability to discriminate between vegetation types [45,46].

### 4.2. Driving Factor Analysis of Spatial Patterns in AGB in the Beilu River Basin

We used the geodetector method to analyze the impacts of 10 factors on the spatial distribution of AGB in the alpine grassland within the Beilu River Basin. Vegetation type emerged as the dominant factor, with a q value of 0.63, indicating a high explanatory ability for AGB variation in the study area. This suggests significant AGB variability among different grassland types, attributed to variations in vegetation cover and community composition, which is supported by field sample data (Table 1). The second most important factor was surface soil moisture, with a q value of 0.52, implying its critical role in grassland ecosystem distribution and composition and its regulation of the AGB [59,60]. Among climatic factors, annual precipitation and mean annual air temperatures (MAATs) had q-values of 0.22 and 0.15, respectively, suggesting their influence on AGB, with annual precipitation being more significant in regards to grassland growth conditions in the Qinghai–Xizang Plateau. This is in agreement with most of the current studies that concluded that the annual precipitation, especially precipitation in the warm season, can directly affect the AGB of the growing season [1,58,61]. The SCDs had the lowest q value (0.04), suggesting a minor effect on the AGB spatial pattern.

The Beilu River Basin is indeed characterized by permafrost, which significantly influences the alpine grassland ecosystem. The permafrost-affected alpine grasslands of the Tibetan Plateau have been studied extensively [28,29,62,63,64], with soil texture and microbial communities proven to play a crucial role in the carbon biogeochemistry of these terrestrial ecosystems. Our finding that ALT had a high q-value of 0.38 suggests a strong explanatory power in regards to AGB spatial distribution patterns, as evidenced by the fact that the AGB increases as the thickness of the active layer decreases. This is likely due to a smaller ALT corresponding to a higher permafrost top plate, favoring soil moisture preservation in the active layer, as indicated by the enhanced interaction (0.6) between ALT and soil moisture, as shown in Figure 8. On the other hand, the MAGT had a low q-value of 0.14, suggesting a weak influence on AGB. This could be because while MAGT affects the overall thermal state of the permafrost, it might not directly influence plant growth, hence exerting a smaller impact on AGB. These results indeed imply that the interaction between the AGB and the permafrost characteristics is complex. Further research and comprehensive field data would be needed to fully understand these complex interactions.

The topographic factors (DEM, aspect, and slope) exhibited low q-values ranging from 0.14 to 0.18, which suggested a weak ability to explain the AGB variation. This could be attributed to the scale effect of the topographic data resolution (1 km) used in this study [30,58]. This 1 km spatial resolution may be adequate for alpine steppe or alpine desert steppe areas with relatively small topographic variation, but might not capture the fine-scale variation in more rugged regions, such as alpine meadows or swamp meadows, in the mid- and high-mountainous areas [50].

## 5. Conclusions

In this study, a random forest (RF) model was developed using 9 vegetation indices (VIs) and 140 field-measured aboveground biomass (AGB) data points as input features, implemented in the Google Earth Engine (GEE) platform, to enhance AGB estimation in the alpine grasslands of the Beilu River Basin in the Qinghai–Xizang Plateau permafrost zone. The study resulted in the generation of spatial distribution maps with a 30-m resolution, providing detailed insights into the AGB distribution and identifying and quantifying the key drivers of AGB in these alpine grassland ecosystems using geodetector technology. The key findings of this study are as follows:

(1) The RF model incorporating NDVI, EVI, SAVI, DVI, NBR1, and NBR2 demonstrated the highest performance, achieving an *R*^2^ value of 0.7956 and an *RMSE* of 66.47 g/m^2^. This indicates that SAVI and NBR2 are crucial variables to consider in regards to AGB estimation for alpine grasslands in the Qinghai–Xizang Plateau.
(2) The spatial distribution of AGB in the study area’s alpine grasslands exhibits higher values in the southern regions and lower values in the northern regions. The AGB values for various grassland types within the study area are notably higher than those of similar grasslands in the Qinghai–Xizang Plateau, with alpine meadows reaching an AGB of 285 g/m^2^ and alpine steppe showing values of 204 g/m^2^.
(3) Geodetector analysis indicated that grassland type, soil moisture, and active layer thickness (ALT) are more significant factors in explaining the spatial distribution of AGB than are climatic (MAAT, precipitation, and SCDs) and topographic factors. This suggests that these variables should be prioritized in future studies and management strategies to better understand and predict AGB dynamics in alpine grassland ecosystems.

## Figures and Tables

**Table 1:** Characteristics of the vegetation biomass of the Beilu River Basin.

Grassland Type	Number of Samples	Vegetation Community	Average Vegetation Cover (%)	Average AGB (g/m^2^)	Standard Deviation
Alpine swamp meadow	22	*Kobresia pygmaea* *Kobresia littledalei*	77	418.9	93.13
Alpine meadow	37	*Kobresia pygmaea* *Kobresia littledalei*	64	313.85	85.41
Alpine steppe	43	*Carex moorcroftii* *Stipa purpurea* *Leontopodium pusillum* *Littledalea racemosa* *Poa pratensis* *Androsace tangulashanensis*	35	234.82	70.95
Alpine desert steppe	26	*Stipa purpurea* *Short spike fescue* *Leontopodium pusillum* *Poa pratensis* *Myricaria prostrata*	18	106.87	56.85
Alpine desert	12	*Arenaria qinghaiensis* *Androsace tapete* *Rhodiola* *Saussurea tangutica*	1.5	27.76	15.49

**Table 2:** The vegetation indices used in the RF model.

Vegetation Index	Formula	References
NDVI	$\frac{NIR-Red}{NIR+Red}$	[35,36]
EVI	*G*× $\frac{NIR-Red}{NIR+C_{1}\times Red-C_{2}\times Blue+L}$ *G* = 2.5; $C_{1}$ = 6; $C_{2}$ = 7.5; *L* = 1	[37,38]
SAVI	$\frac{NIR-Red}{NIR+Red+L}\times \left(1+L\right)$ *L* = 0.5	[39,40]
DVI	NIR−Red	[41,42]
RVI	$\frac{NIR}{Red}$	[16,43]
GNDVI	$\frac{NIR-Green}{NIR+Green}$	[44,45]
GRVI	$\frac{Green-Red}{Green+Red}$	[36,46]
NBR	$\frac{NIR-SWIR2}{NIR+SWIR2}$	[47]
NBR2	$\frac{SWIR1-SWIR2}{SWIR1+SWIR2}$	[48]

**Table 3:** The performance of the AGB estimation models for different VIs for the training set.

Model	VIs	*R* ^2^	*RMSE*
1	NDVI	0.6081	87.58
2	EVI	0.6124	88.21
3	NDVI, EVI	0.7005	79.15
4	NDVI, EVI, NBR2	0.7202	75.02
5	NDVI, EVI, SAVI	0.7156	73.5
6	NDVI, EVI, SAVI, NBR2	0.7546	71.84
7	NDVI, EVI, SAVI, NBR1, NBR2	0.7695	68.54
8	NDVI, EVI, SAVI, DVI, NBR1, NBR2	0.7956	66.47
9	NDVI, EVI, SAVI, DVI, RVI, NBR1, NBR2	0.7787	65.47
10	NDVI, EVI, SAVI, DVI, RVI, GRVI, NBR1, NBR2	0.7584	69.32
11	NDVI, EVI, SAVI, DVI, RVI, GNDVI, GRVI, NBR1, NBR2	0.7285	71.64

**Table 4:** Optimal discretization methods for 10 factors.

Variable	Optimal Method	Number of Strata
Precipitation	sd	10
MAAT	sd	10
ALT	quantile	10
DEM	natural	10
MAGT	geometric	9
Soil moisture	quantile	10
Slope	quantile	10
SCDs	geometric	10
Aspect	manual	8
Vegetation types	manual	6

## Data Availability

Data is contained in the article.

## References

[1] Wang Y., Lv W., Xue K., Wang S., Zhang L., Hu R., Zeng H., Xu X., Li Y., Jiang L. (2022). Grassland changes and adaptive management on the Qinghai–Tibetan Plateau. Nat. Rev. Earth Environ..

[2] Akiyama T., Kawamura K. (2007). Grassland degradation in China: Methods of monitoring, management and restoration. Grassl. Sci..

[3] CEOS Land Product Validation Subgroup. https://lpvs.gsfc.nasa.gov/AGB/AGB_home.html.

[4] John R., Chen J., Giannico V., Park H., Xiao J., Shirkey G., Ouyang Z., Shao C., Lafortezza R., Qi J. (2018). Grassland canopy cover and aboveground biomass in mongolia and inner mongolia: Spatiotemporal estimates and controlling factors. Remote Sens. Environ..

[5] Xia J., Ma M., Liang T., Wu C., Yang Y., Zhang L., Zhang Y., Yuan W. (2018). Estimates of grassland biomass and turnover time on the tibetan plateau. Environ. Res. Lett..

[6] Yu L., Zhou L., Liu W., Zhou H. (2010). Using Remote Sensing and GIS Technologies to Estimate Grass Yield and Livestock Carrying Capacity of Alpine Grasslands in Golog Prefecture, China. Pedosphere.

[7] Anaya J., Chuvieco E., Palacios-Orueta A. (2009). Aboveground biomass assessment in Colombia: A remote sensing approach. For. Ecol. Manag..

[8] Claverie M., Demarez V., Duchemin B., Hagolle O., Ducrot D., Maraissicre C., Dejoux J., Huc M., Keravec P., Béziat P. (2012). Maize and sunflower biomass estimation in southwest France using high spatial and temporal resolution remote sensing data. Remote Sens. Environ..

[9] Punalekar S., Verhoef A., Quaife T., Humphries D., Bermingham L., Reynolds C. (2018). Application of sentinel-2a data for pasture biomass monitoring using a physically based radiative transfer model. Remote Sens. Environ..

[10] Forkuor G., Zoungrana J.-B.B., Dimobe K., Ouattara B., Vadrevu K.P., Tondoh J.E. (2020). Above-ground biomass mapping in west african dryland forest using sentinel-1 and 2 datasets-a case study. Remote Sens. Environ..

[11] Liu S., Cheng F., Dong S., Zhao H., Hou X., Wu X. (2017). Spatiotemporal dynamics of grassland aboveground biomass on the qinghai-tibet plateau based on validated modis ndvi. Sci. Rep..

[12] Gao X., Dong S., Li S., Xu Y., Liu S., Zhao H., Yeomans J., Li Y., Shen H., Wu S. (2020). Using the random forest model and validated modis with the field spectrometer measurement promote the accuracy of estimating aboveground biomass and coverage of alpine grasslands on the qinghai-tibetan plateau. Ecol. Indic..

[13] Wang J., Xiao X., Bajgain R., Starks P., Steiner J., Doughty R., Chang Q. (2019). Estimating leaf area index and aboveground biomass of grazing pastures using Sentinel-1, Sentinel-2 and Landsat images. ISPRS J. Photogramm. Remote Sens..

[14] Li C., Zhou L., Xu W. (2021). Estimating Aboveground Biomass Using Sentinel-2 MSI Data and Ensemble Algorithms for Grassland in the Shengjin Lake Wetland, China. Remote Sens..

[15] Sibanda M., Mutanga O., Rouget M. (2016). Comparing the spectral settings of the new generation broad and narrow band sensors in estimating biomass of native grasses grown under different management practices. GISci. Remote Sens..

[16] Ren H., Zhou G. (2019). Estimating green biomass ratio with remote sensing in arid grasslands. Ecol. Indic..

[17] Xia J., Liu S., Liang S., Chen Y., Xu W., Yuan W. (2014). Spatio-Temporal Patterns and Climate Variables Controlling of Biomass Carbon Stock of Global Grassland Ecosystems from 1982 to 2006. Remote Sens..

[18] Yang Y., Fang J., Pan Y.D., Ji C. (2009). Aboveground biomass in Tibetan grasslands. J. Arid Environ..

[19] Mutanga O., Skidmore A.K. (2004). Narrow band vegetation indices overcome the saturation problem in biomass estimation. Int. J. Remote Sens..

[20] Zhao D., Arshad M., Wang J., Triantafilis J. (2021). Soil exchangeable cations estimation using vis-nir spectroscopy in different depths: Effects of multiple calibration models and spiking. Comput. Electron. Agric..

[21] Wang Y., Wu G., Deng L., Tang Z., Wang K., Sun W., Shangguan Z. (2017). Prediction of aboveground grassland biomass on the Loess Plateau, China, using a random forest algorithm. Sci. Rep..

[22] Yu R., Yao Y., Wang Q., Wan H., Xie Z., Tang W., Zhang Z., Yang J., Shang K., Guo X. (2021). Satellite-derived estimation of grassland aboveground biomass in the three-river headwaters region of china during 1982–2018. Remote Sens..

[23] Liang T., Yang S., Feng Q., Liu B., Zhang R., Huang X., Xie H. (2016). Multi-factor modeling of above-ground biomass in alpine grassland: A case study in the Three-River Headwaters Region, China. Remote Sens. Environ..

[24] Yang Y., Dou Y., An S. (2017). Environmental driving factors affecting plant biomass in natural grassland in the Loess Plateau, China. Ecol. Indic..

[25] Ge J., Hou M., Liang T., Feng Q., Meng X., Liu J., Bao X., Gao H. (2022). Spatiotemporal dynamics of grassland aboveground biomass and its driving factors in North China over the past 20 years. Sci. Total Environ..

[26] Kaveh N., Ebrahimi A., Asadi E. (2023). Comparative analysis of random forest, exploratory regression, and structural equation modeling for screening key environmental variables in evaluating rangeland above-ground biomas. Ecol. Inform..

[27] Zhang X., Sheng Y., Li J., Wu J., Chen J., Cao Y. (2012). Changes of alpine ecosystem along the ground temperature of permafrost in the source region of Datong River in the northeastern Qinghai-Tibet Plateau. J. Food Agric. Environ..

[28] Zhou Z., Yi S., Chen J., Ye B., Sheng Y., Wang G., Ding Y. (2015). Responses of alpine grassland to climate warming and permafrost thawing in two basins with different precipitation regimes on the Qinghai-Tibetan Plateaus. Arct. Antarct. Alp. Res..

[29] Zhang Y., Zhou T., Shi P., Liu X., Yu P., Luo H., Zhou P., Xu Y. (2023). Modeling of grassland biomass and evaluation of uncertainties caused by differences in frozen soil type on the Qinghai Plateau. Theor. Appl. Climatol..

[30] Zou D., Zhao L., Sheng Y., Chen J., Hu G., Wu T., Wu J., Xie C., Wu X., Pang Q. (2017). A new map of permafrost distribution on the Tibetan Plateau. Cryosphere.

[31] Xu Y., National Earth System Science Data Center, National Science & Technology Infrastructure of China (2023). Annual Mean Temperature Data at 1 km Resolution in China (1982–2022). http://www.geodata.cn/data/datadetails.html?dataguid=67669514169502&docid=209.

[32] Xu Y., National Earth System Science Data Center, National Science & Technology Infrastructure of China (2023). Annual Precipitation Data at 1 km Resolution in China (1982–2022). http://www.geodata.cn/data/datadetails.html?dataguid=113786088533256.

[33] Li X., Li L., Shi F., Su W., Xiao J., Li H. (2021). Remote Sensing Monitoring Data Set of Snow Days in Qinghai Province from 2002 to 2021. http://www.ncdc.ac.cn/portal/metadata/f4625268-69d4-4210-be1f-c0d1ded375e4.

[34] Ran Y., Li X., Che T., Wang B., Cheng G. (2022). Current State and Past Changes in Frozen Ground at the Third Pole. National Tibetan Plateau/Third Pole Environment Data Center. https://data.tpdc.ac.cn/zh-hans/data/ade493c8-3692-4871-bcb4-a4fabaef04a9.

[35] Gamon J.A., Field C.B., Goulden M.L., Griffin K.L., Hartley A.E., Joel G., Penuelas J., Valentini R. (1995). Relationships between NDVI, canopy structure, and photosynthesis in three Californian vegetation types. Ecol. Appl..

[36] Tucker C.J. (1979). Red and photographic infrared linear combinations for monitoring vegetation. Remote Sens. Environ..

[37] Liu H., Huete A. (1995). A feedback based modification of the ndvi to minimize canopy background and atmospheric noise. IEEE Trans. Geosci. Remote Sens..

[38] Shen M., Tang Y., Klein J., Zhang P., Gu S., Shimono A., Chen J. (2008). Estimation of aboveground biomass using in situ hyperspectral measurements in five major grassland ecosystems on the tibetan plateau. J. Plant Ecol..

[39] Huete A.R. (1988). A soil-adjusted vegetation index (savi). Remote Sens. Environ..

[40] Fu G., Shen Z.X. (2016). Environmental humidity regulates effects of experimental warming on vegetation index and biomass production in an alpine meadow of the northern tibet. PLoS ONE.

[41] Yan F., Wu B., Wang Y. (2013). Estimating aboveground biomass in mu us sandy land using landsat spectral derived vegetation indices over the past 30 years. J. Arid Land.

[42] Wang G., Liu S., Liu T., Fu Z., Yu J., Xue B. (2019). Modelling above-ground biomass based on vegetation indexes: A modified approach for biomass estimation in semi-arid grasslands. Int. J. Remote Sens..

[43] Jordan C.F. (1969). Derivation of leaf-area index from quality of light on the forest floor. Ecology.

[44] Gitelson A., Kaufman Y., Merzlyak M. (1996). Use of a green channel in remote sensing of global vegetation from EOS-MODIS. Remote Sens. Environ..

[45] Yawata K., Yamamoto T., Hashimoto N., Ishida R., Yoshikawa H. (2019). Mixed model estimation of rice yield based on NDVI and GNDVI using a satellite image. Remote Sens. Agric. Ecosyst. Hydrol. XXI.

[46] Yin G., Verger A., Descals A., Filella I., Peñuelas J. (2022). A Broadband Green-Red Vegetation Index for Monitoring Gross Primary Production Phenology. J. Remote Sens..

[47] Key C., Benson N. (1999). Measuring and remote sensing of burn severity. Proceedings Joint Fire Science Conference and Workshop.

[48] Key C., Benson N. (2006). Landscape assessment (LA). FIREMON: Fire Effects Monitoring and Inventory System.

[49] Jin Z., Feng Q., Wang R., Liang T. (2022). A study of grassland aboveground biomass on the Tibetan. Acta Prataculturae Sin..

[50] Zhang H., Tang Z., Wang B., Kan H., Sun Y., Qin Y., Meng B., Li M., Chen J., Lv Y. (2023). A 250m annual alpine grassland AGB dataset over the Qinghai-Tibetan Plateau (2000–2019) based on in-situ measurements, UAV images, and MODIS Data. Earth Syst. Sci. Data..

[51] Chen Z., Wang H., Wang J., Shi H., Liu H., He J. (2021). Estimation on seasonal dynamics of alpine grassland aboveground biomass using phenology camera-derived NDVI. Chin. J. Plant Ecol..

[52] Zhu Q., Chen H., Peng C., Liu J., Piao S., He J.-S., Wang S., Zhao X., Zhang J., Fang X. (2023). An early warning signal for grassland degradation on the Qinghai-Tibetan Plateau. Nat. Commun..

[53] Zhang H., Tang Z., Wang B., Meng B., Qin Y., Sun Y., Lv Y.Y., Zhang J., Yi S. (2022). A non-destructive method for rapid acquisition of grassland aboveground biomass for satellite ground verification using UAV RGB images. Glob. Ecol. Conserv..

[54] Lussem U., Bolten A., Menne J., Gnyp M., Schellberg J., Bareth G. (2019). Estimating biomass in temperate grassland with high resolution canopy surface models from UAV-based RGB images and vegetation indices, *J*. Appl. Remote Sens..

[55] Meng B., Yi S., Liang T., Yin J., Sun Y. (2020). Modeling alpine grassland above ground biomass based on remote sensing data and machine learning algorithm: A case study in the east of Tibetan Plateau, China. IEEE J. Select. Top. Appl. Earth Obs. Remote Sens..

[56] Zhang B., Zhang L., Xie D., Yin X., Liu C., Liu G. (2016). Application of Synthetic NDVI Time Series Blended from Landsat and MODIS Data for Grassland Biomass Estimation. Remote Sens..

[57] Huete A., Justice C., Liu H. (1994). Development of vegetation and soil indices for MODIS-EOS. Remote Sens. Environ..

[58] Jiang Y., Tao J., Huang Y., Zhu J., Tian L., Zhang Y. (2015). The spatial pattern of grassland aboveground biomass on Xizang Plateau and its climatic controls. J. Plant Ecol..

[59] Wei P., Pan X., Xu L., Hu Q., Zhang X., Guo Y., Shao C., Wang C., Li Q., Yin Z. (2019). The effects of topography on aboveground biomass and soil moisture at local scale in dryland grassland ecosystem, China. Ecol. Indic..

[60] Zhang J., Fang S., Liu H. (2022). Estimation of alpine grassland above-ground biomass and its response to climate on the Qinghai-Tibet Plateau during 2001 to 2019. Glob. Ecol. Conserv..

[61] Zeng N., Ren X., He H., Zhang L., Zhao D., Ge R., Li P., Niu Z. (2019). Estimating grassland aboveground biomass on the Tibetan Plateau using a random forest algorithm. Ecol. Indic..

[62] Yue G., Zhao L., Wang Z., Zhang L., Zou D., Niu L., Zhao Y., Qiao Y. (2017). Spatial variation in biomass and its relationships to soil properties in the permafrost regions along the Qinghai-Tibet Railway. Environ. Eng. Sci..

[63] Mu C., Li L., Zhang F., Li Y., Xiao X., Zhao Q., Zhang T. (2018). Impacts of permafrost on above-and belowground biomass on the northern Qinghai-Tibetan Plateau. Arct. Antarct. Alp. Res..

[64] Tian L., Zhao L., Wu X., Hu G., Fang H., Zhao Y., Sheng Y., Chen J., Wu J., Li W. (2019). Variations in soil nutrient availability across Tibetan grassland from the 1980s to 2010s. Geoderma.

